# Prolonged conservative treatment or 'early' surgery in sciatica caused by a lumbar disc herniation: rationale and design of a randomized trial [ISRCT 26872154]

**DOI:** 10.1186/1471-2474-6-8

**Published:** 2005-02-11

**Authors:** Wilco C Peul, Hans C van Houwelingen, Wilbert B van der Hout, Ronald Brand, Just AH Eekhof, Joseph ThJ Tans, Ralph TWM Thomeer, Bart W Koes

**Affiliations:** 1 Department of Neurosurgery, Leiden University Medical Center, PO Box 9600, 2300 RC Leiden, The Netherlands; 2 Department of Medical Statistics, Leiden University Medical Center, Leiden, The Netherlands; 3 Department of Medical Decision Analysis, Leiden University Medical Center, Leiden, The Netherlands; 4 Department of General Practice, Leiden University Medical Center, Leiden, The Netherlands; 5 Department of Neurology, Medical Center Haaglanden, The Hague, The Netherlands; 6 Department of General Practice, University Medical Center Rotterdam (Erasmus MC), PO Box 1736 Rotterdam, The Netherlands

## Abstract

**Background:**

The design of a randomized multicenter trial is presented on the effectiveness of a prolonged conservative treatment strategy compared with surgery in patients with persisting intense sciatica (lumbosacral radicular syndrome).

**Methods/design:**

Patients presenting themselves to their general practitioner with disabling sciatica lasting less than twelve weeks are referred to the neurology outpatient department of one of the participating hospitals. After confirmation of the diagnosis and surgical indication MRI scanning is performed. If a distinct disc herniation is discerned which in addition covers the clinically expected site the patient is eligible for randomization. Depending on the outcome of the randomization scheme the patient will either be submitted to prolonged conservative care or surgery. Surgery will be carried out according to the guidelines and between six and twelve weeks after onset of complaints. The experimental therapy consists of a prolonged conservative treatment under supervision of the general practitioner, which may be followed by surgical intervention in case of persisting or progressive disability. The main primary outcome measure is the disease specific disability of daily functioning. Other primary outcome measures are perceived recovery and intensity of legpain. Secondary outcome measures encompass severity of complaints, quality of life, medical consumption, absenteeism, costs and preference. The main research question will be answered at 12 months after randomization. The total follow-up period covers two years.

**Discussion:**

Evidence is lacking concerning the optimal treatment of lumbar disc induced sciatica. This pragmatic randomized trial, focusses on the 'timing' of intervention, and will contribute to the decision of the general practictioner and neurologist, regarding referral of patients for surgery.

## Background

One of the greatest advantages of publishing the design of a randomized controlled trial (RCT) before results are available is the accessibility to criticism of the methodological quality irrespective of the results. Firstly the scientific reader must be enabled to search for epidemiological shortcomings when the results differ from the expected outcome as compared to results in line with one's expectations. Secondly, it is possible to more extensively elaborate the background and rationale of the research question, the study population, the chosen treatments and outcome measures, as compared to publications describing the trial results. Thirdly, but not less important, publishing the design of a RCT is instrumented in preventing publication bias in subsequent meta-analyses. Studies with non-significant results are less likely to be published than those with significant results [1,2]. It is a considerable loss for data pooling that unpublished trial results are omitted. After pre-publishing the study design even unpublished data can be used in a systematic review, since these can be required from the study group. This article describes the rationale and parallel group design of a RCT in which the optimal timing of disc surgery for sciatica will be investigated.

The lumbosacral radicular syndrome (LSRS or LRS; also called sciatica) is typically characterized by radiating pain in the dermatome of a lumbar or sacral spinal nerve root. Occasionally more than one root is involved. Contained in the syndrome pain may be accompanied with lumbar fixation, reflex abnormalities motor and sensory disturbances. In diagnosis includes stenosis of the spinal and/or root canal, infection, multiple sclerosis, autoimmune or metabolic neuropathy, and tumour. This study will be restricted to herniations at the lowest three lumbar disc levels, since these represent the most common sites. In the vast majority of cases LSRS is the result of a herniated disc. In the Netherlands annually between 60,000 and 75,000 new cases of LSRS are diagnosed by the General Practitioner (GP) [3]. The presumed direct medical costs of treatment of LSRS are € 133 million each year [4]. Most of these costs are attributable to in-hospital treatment; only a small portion is incurred by GP's or physiotherapists (€ 3.2 million). In a study, performed in 1988, more than 11.000 patients were operated in the Netherlands and this frequency did not change in the past years [4,5]. The combined direct and indirect costs are estimated to be € 1,2 billion per year [6]. The indirect costs are considerable due to the high rate of production loss caused by sciatica.

The natural history of LSRS is in general favourable. In 60–80 percent of patients, the leg pain decreased or disappeared within 6–12 weeks after onset [7-9,51]. These patients no longer experienced problems at work or in their private lives after three months. The minority with lasting complaints beyond three months further decreases with time. At one year only a small proportion of herniated discs continues to produce discomfort and disability. At present it is not possible to identify these latter groups of patients in an early stage of their disease by means of intensity of pain, neurological deficit, root irritation signs, or diagnostic imaging. For this reason it is not helpful to perform early diagnostic imaging (CT or MRI), unless a disease entity different from disc herniation is considered. After the indication for surgery has been set diagnostic imaging is helpful in defining the exact site of disc herniation and its anatomical relationship with the nerve root involved.

Since the first publication on lumbar disc surgery by Mixter and Barr [17] many studies have demonstrated the success of surgery for the treatment of LSRS. Unfortunately only a few prospective studies investigated the difference in outcome between surgical and conservative care [7,8,18-22]. The published treatment results vary as much as the frequency of reported complications and the recurrence rate.

The only study, which compared surgery with conservative care directly in a RCT, was performed by Weber more than 20 years ago [7,8]. He found better results for surgery at one-year follow-up. At four and ten years follow-up the results of surgical and conservative care no longer differed. Being the only published RCT comparing surgical and conservative care, this study regrettably carries some important methodological flaws in both design and outcome measures when compared to today's epidemiological standard rules [23]. One of the main shortcomings is the exclusion of patients, who do have an indication for surgery because of "intolerable" pain. Those are the current patients who ask for surgery and are not comparable to the randomized population of Weber. Therefore it is impossible to extrapolate and generalize these results to the treatment policy of today.

Since 1983 a few cohort studies have been published on non-surgical treatment of patients with at least six weeks of leg pain with good short-term results at one-year follow-up [25,22]. These studies also suffer from methodological flaws. The only conclusion that can be drawn from these reports and the study of Weber is that the policy of prolonged conservative care can be effective, as a result of the favourable natural course of LSRS. Epidemiological and clinical studies have shown that most lumbar disc protrusions resolve spontaneously with the elapse of time [15,16]. Another finding is that prolonged conservative care appears safe and without complications if the patient remains active. Recent population based studies however state that the natural history is not favourable at all [50].

Whether particular demographic findings, symptoms, physical signs and/or MRI findings either separately or combined do have prognostic value has not been investigated scientifically yet. It would be of great value if one were able to identify early in the course of the disease those patients who will have an unfavourable outcome without surgery.

In spite of the known favourable natural course the surgical rate in the Netherlands is quite high [10]. We perform six times as many lumbar discectomies compared to Scotland, four times the number in England and two times the number in Sweden. In the latter study comparing 12 Western countries the United States is the only country where more operations are performed for the indication LSRS. There are no substantial differences in the incidence of this disease in the countries mentioned that can explain the difference in surgical rates. There is no indication [6] that the surgical rate has changed under influence of the consensus reports [11,12,11]. Actually change was not likely to occur because the published guidelines were representative for daily practice and normal care before 1996 in the Netherlands. With respect to the indications for and timing of surgery no evidence in the literature is available to either support or contradict these guidelines. These guidelines were produced after agreement between all medical (sub-) disciplines involved in the care for patients with LSRS. Our high surgical rate, as contradictory as it may seem, may reflect good clinical practice.

Because of the observation that most people recover from their complaints in the first 6–8 weeks [9,51] this period of persistent radicular leg pain is considered a good indication for surgery in the Netherlands. Although there is consensus that surgery is only offered in case of persistent pain, the timing of this treatment seems to depend on local production capacity and patient and doctor preferences rather than on evidence-based practice. This lack of evidence for the timing of surgery after the 6–8 week period explains the large variations in daily practice. Exact data on the problems associated with surgery, such as surgical failure, recurrent disc herniation and adverse effects are limited. This is one of the reasons that in some regions surgery will only be carried out after a period of 3–6 months of LSRS. [14].

It is not known whether the relative high rate of disc surgery in the Netherlands is cost-effective or not, compared to other countries [15,16].

In summary, consensus is missing on the preferred timing of disc surgery, due to insufficient evidence that a prolonged conservative care strategy is effective. More insight is needed into the potential short-term effects of a relative early surgery strategy, as compared to an extended wait-and-see period. In particular the effects on the return to work or resumption of previous daily activities as well as the complications of both strategies have not yet been investigated.

The main goal of this comparative study is to investigate whether the completion of a 6–12 weeks period of lasting radicular pain constitutes a solid indication for surgery and is superior to prolonged conservative care. A secondary goal is to identify possible subgroups of patients who will substantially benefit from one of the proposed treatment strategies. The cost-effectiveness results will be a trade-off between a quicker relief of leg pain in the surgery group versus the advantage of lower costs and avoiding the negative effects of surgery in the conservatively treated group. The difference in disease related quality of life depends on the duration of persisting pain and disability after randomization in the prolonged conservative care group.

This study to investigate this scientific gap in our understanding of the effectiveness of surgery for LSRS is in line with a recommendation by the Dutch Health Council in 1999 to the Minister of Health [4] and the current Cochrane Review [15,16].

The results of this trial will lead to a more rational use of the existing guidelines if the hypothesis is rejected. If the latter is accepted and prolongation of the conservative treatment policy is more cost-effective than surgery after 6–12 weeks, the current guidelines for the timing of surgery need correction.

## Methods/design

**Figure 1:**
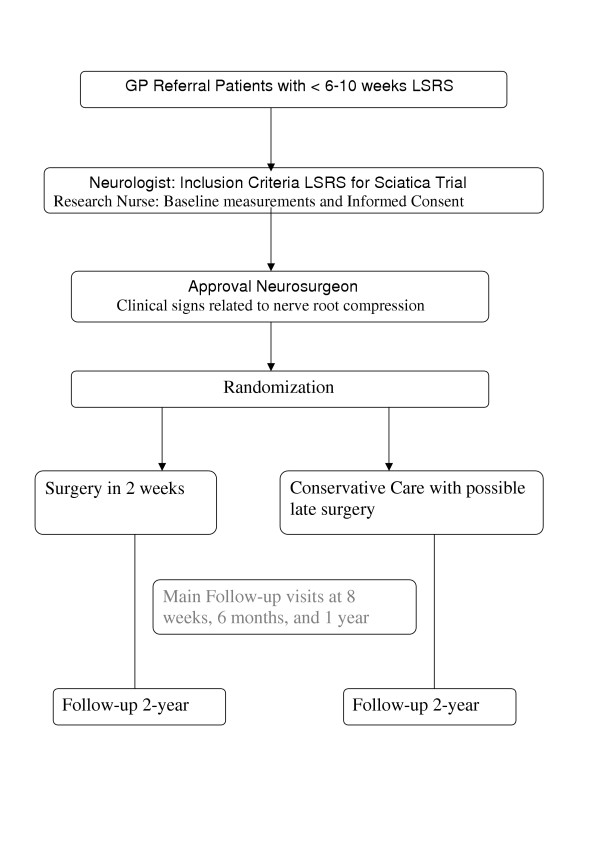
Flow chart of the Sciatica Trial

To answer the main research question the investigators propose to conduct a multi-centre comparative randomized clinical trial with parallel group design. The main research question will be answered after a follow-up of six months (Figure 1). The complete follow-up will last two years. The multi-centre design is necessary to collect enough patients in two years. The Medical Ethics Committee of all participating hospitals approved the study protocol.

### Patients

All patients between 18 and 65 years with sciatica of less than 12 weeks duration are eligible for this study. Because of the multi-centre (15 hospitals) design the patients in a large region in the western part of the Netherlands can be included in this trial if they meet the in- and exclusion criteria (Table 1). Because these are the only hospitals, which treat lumbar disc herniations in this area, included patients will reflect a representative population treated in primary and secondary care. Inclusion of patients will be started after a visit to the neurological outpatient clinics. Randomization will start after at least 6 weeks persistent disabling pain in the dermatome of the leg served by the L4, L5 or S1 root. All 1100 GP's involved will be informed about this study and receive information about developments and the results of the trial. They will refer patients within the first 6–12 weeks after onset sciatica.

**Table 1:** Selection criteria for trial eligibility

**Inclusion criteria:**
• Age 18–65 yr.
• Persistent radicular pain in the L4, L5 or S1 dermatome with or without mild neurological deficit
• Severe disabling leg pain of 6–12 weeks duration
• Evidence of a unilateral disc herniation confirmed on MRI
• Sufficient knowledge of Dutch language
• Informed consent

**Exclusion criteria:**
• Cauda equina syndrome or severe paresis (MRC<3)
• Complaints of a lumbosacral radicular syndrome in the same dermatome within the past 12 months
• A history of unilateral disc surgery on the same level
• Spinal canal stenosis
• Degenerative or lytic spondylolisthesis
• Pregnancy
• "Severe life-threatening" or psychiatric illness
• Planned (e)migration to another country in the year after randomization

During the first visit to the neurological outpatient clinic the patient's history will be taken and a standardized neurological examination will be performed. During this visit the neurologist will inform the patient on the cause and course of a lumbosacral radicular syndrome and convey the doubt regarding the timing of surgery for this condition. The study will be explained to the patient and in case of a positive reaction an appointment is made to meet one of the research nurses as soon as possible.

Preferably the study MRI scans will be performed after informed consent during the first visit to the research nurse. Because the patient needs some time to consider participation a second visit will be planned at least two days after the first visit to the outpatient clinic. The research nurse will give all extra information needed to understand the trial and will ask the patient if he/she agrees to be randomized. Informed by the radiologist and surgeon, the research nurse will only randomize the patient during the third visit if the MRI confirms the presence of unilateral disc herniation and the patient is eligible according to the inclusion and exclusion criteria. The patient will not be aware of detailed MRI data. The radiologist and neurosurgeon independently using a standardized Case Record Form (CRF) will register the MRI findings. The MRI will be performed according to a standardized protocol and including Gadolinium series for the intended subgroup analysis.

### Treatment allocation

Patients will randomly be allocated to either surgery within 1–2 weeks or prolonged conservative treatment by their GP. Patients, their doctors and research nurses can obviously not be blinded for the allocated treatment. Blinding of the outcome measurements is not possible, due to the fact that mainly self-reported outcomes are used. A randomization list is prepared for every participating hospital. Permuted blocks of random number patients are formed to ensure near-equal distribution of patients over the two randomization arms in the hospitals. Using random number tables generates the random sequence of the permuted blocks. The data manager, who is not involved in the selection and allocation of patients will prepare coded, sealed envelopes containing the treatment allocation. During the second patient visit the research nurse will open the envelope together with the patient and appointments will be made for the allocated treatment, either surgery or referral back to the GP, to ensure that treatment is started as soon as possible after randomization. This will be done after checking all the criteria and especially the persistence of pain with disability in daily functioning. A letter about the allocated treatment arm informs all caregivers. Although the principal investigator will not include and operate upon trial patients he may be biased with a preference for surgery, which could theoretically influence analysis. Therefore the principal investigator is blinded for the allocated treatment. As he is not involved in treatment of the study population blinding during later analysis is only possible after blinding during the randomization and follow-up period.

### Interventions

After randomization two groups of patients will exist.

*Group A*; the surgically treated patients and *group B; *the conservatively managed patients.

#### Surgical treatment

(A) will be performed in the conventional manner with microscope or loupe magnification. The investigators prefer the standard surgical approach because the other (minimally invasive) surgical approaches have limited indications, are not more cost-effective, and have a long learning curve. During the transflaval approach care is undertaken to minimize bony removal and on the other hand to prevent overstretching of the compromised nerve root. In addition to removal of herniated disc material as much as possible nuclear material will be removed with pituitary forceps, curettes and rongeurs in order to prevent recurrence. The participating treating doctors are 2 orthopaedic- and 12 neurosurgeons with large experience in the standard approach with loupe magnification or microscope. A standardized CRF will register the findings of the surgeon and the herniated disc material will be investigated histologically for granular infiltration.

Surgery will take place as soon as possible and within a maximum of two weeks after randomization. Hospital admission will be 2–7 days, including the day of surgery. During the immediate post-operative period the patients will be mobilised with the help of a physiotherapist. At home guidance is confirmed by their own physiotherapist. The frequency will be 2 times a week for 8 weeks.

#### Conservative management

(B) will be conducted by the general practitioner (GP) or neurologist when necessary. The GP will provide ample information about the favourable prognosis of LSRS. The treatment of LSRS is aimed primarily at pain relief and maintenance/restoration of normal day-to-day activities. Unfortunately, the effect of giving information and counselling has not been studied specifically among LSRS patients. However, various studies have evaluated the effect of such support for people suffering from other pain syndromes [24]. Inferences can reasonably be made from the findings of these studies. Hence, it may be assumed that adequate and unambiguous information about what is wrong (the nature of the condition) and what the patient can expect (the prognosis), together with trustworthy counselling can reduce the anxiety and uncertainty felt by the patients and thus ease the pain [12]. The GP's will encourage the patients to continue with normal day-to-day activities in so far as possible. When necessary analgesic medication can be prescribed according to the guidelines. The GP will advise the patients to stay active and if possible return to work and/or their leisure activities.

After the first consultation the GP will make a follow-up schedule. During the next visit the patient and doctor will look at the changes since the first visit to determine whether there is any improvement in the ability to perform normal activities. The doctor will check the efficacy of the prescribed pain medication and may adjust the dose or sort of analgesics according to the NHG guidelines. In these guidelines paracetamol is the first choice. If not effective, NSAID's (ibuprofen, diclofenac or naproxen) are to be prescribed. Only in the event of severe disabling pain morphine may be given for a restricted period of time. By preference all analgesics should be taken at fixed times of the day rather than on a 'if necessary' basis. If the GP and the patient conclude that there is considerable kinesiophobia because of the fear that the radicular or low back pain will increase, the help of a physiotherapist can be recommended. Guided by the GP (and physiotherapist) the patient will upgrade his or her activities according to the agreed time schedule [25,26]. The guide will be time, not the intensity of the pain. The GP will be free in her/his choice of prescription of medication and referral to physiotherapists. The research nurse will register the conservative management strategy after communication with the responsible GP. In case of progressive neurological deficit or worsening intolerable pain the GP can refer the patient back to the research nurse or neurosurgeon. If, six months after randomization, the patient has still not improved or suffers from intermittent LSRS, surgical treatment will be offered. Some patients will ask for surgery earlier because of worsening drug resistant leg pain. In these cases and in the case of a progressive neurological deficit, surgery will be performed in consultation with the patient. If after maximum conservative treatment and counselling the patient is still not able to cope with the functional disability surgery can be requested. If surgery in these cases is not offered by the study-group the patient does have the right to have a second opinion with an undependable neurosurgeon of another university hospital.

### Outcome assessment

In the LSRS the most common complaints are pain and disability to perform normal daily activities. We will use below described validated outcome parameters, which will be assessed by means of questionnaires. Patients are not informed about their earlier scores. Follow-up examinations by the research nurse will take place 8, 26 and 52 weeks after randomization and the patients will keep a diary (table 2). In between at 2, 4, 12, 38, and 78 and after 104 weeks the main questionnaire (primary outcome measures) will be filled in at home and send to the data centre.

**Table 2:** Data collection and outcome measures

Time in weeks	?	0	2,4	8	12	26	38	52	78	104
Likert	**X**	**X**	**X**	**X**	**X**	**X**	**X**	**X**	**X**	**X**
Neurological examination		**X**		**X**		**X**		**X**		
Severity of complaints (VAS)	**X**	**X**	**X**	**X**	**X**	**X**	**X**	**X**	**X**	**X**
McGill	**X**									
Health Status (SF 36)	**X**			**X**		**X**		**X**		**X**
Functional Status (RDQ)	**X**	**X**	**X**	**X**	**X**	**X**	**X**	**X**	**X**	**X**
EuroQol/VAS Q-of-life	**X**	**X**	**X**	**X**	**X**	**X**	**X**	**X**	**X**	**X**
MRI		**X**							**X**	
Costs	**X**	**X**	**X**	**X**	**X**	**X**	**X**	**X**	**X**	**X**
Prolo	**X**			**X**		**X**		**X**		
Complications		**X**		**X**		**X**		**X**		
Surgery				**X**		**X**		**X**	**X**	**X**
SFBI	**X**	**X**		**X**		**X**		**X**		**X**

#### Primary outcome measures

1) Roland Disability Questionnaire for Sciatica.' This illness-specific 23-item functional assessment questionnaire is frequently used for low back pain and sciatica [38,39]. Scores range from 0 to 23, reflecting a simple unweighted sums of items endorsed by the respondent. Patients with high scores at baseline do have a severe disabling LSRS. To define recovery a difference of at least 11 points from baseline has to be seen [38,17]. The Roland Questionnaire for Sciatica has a documented high level of internal consistency; construct validity, and responsiveness [38,39]. It is the main primary outcome measure in this trial.

2) Perceived recovery.' This is a seven-point Likert scale measuring the perceived recovery, varying from 'completely recovered' to 'worse than ever'. This outcome scale has been used in previous studies and appears to be valid and responsive to change [27]. Next to this global self-assessment a job and hobby specific Likert will be scored. During the intake of the study the patient will be asked to rank their five most important functional disabilities in daily live (work, hobby), which they can use in their own evaluation overall and in separate items.

3) VAS pain in the leg. This parameter will measure the experienced intensity of pain in the leg during the week before visiting the research nurse. Pain will be assessed on a horizontal 100 mm scale varying from 0 mm, 'no pain in the leg', to 100 mm, 'the worst pain ever'. Patients do not see the results of earlier assessments and will score the pain experienced at the visit. [28-32].

#### Secondary outcome measures

1) EuroQol classification system and VAS rating personal health. A cost-utility analysis will be performed using QALY's based on the EuroQol questionnaire, which has been validated in many studies and is easy to fill out [41,42,51]. The EuroQol will be measured twice a week during the first four weeks and at all follow-up moments. Patients describe their general health status using the EuroQol classification system, consisting of 5 questions on mobility, self care, usual activities, pain/discomfort, and anxiety/depression [44]. From the EQ-5D classification system, the EQ-5D utility index will be calculated [43]. This utility measure reflects how the general public values the health status described by the patient, which is preferred for economic evaluations from a societal perspective. Patients also rated their personal health using a visual analog scale (VAS) ranging from worst imaginable health to best imaginable health.

2) Short-Form 36 (SF-36). Quality of life was also assessed using the RAND-36 questionnaire. This is a generic health status questionnaire, which can easily be filled out at home. The questionnaire consists of 36 items on physical and social functioning has 8 domains; 1) physical functioning, 2) physical restrictions, 3) emotional restrictions, 4) social functioning, 5) somatic pain, 6) general mental health, 7) vitality, 8) general health perception. This questionnaire has been used frequently and was validated in studies on low back pathology and surgery [33-37]. From the RAND-36, the SF-6D utility index was calculated. Like the EQ-5D, this SF-6D reflects the general public's valuation of the health described by the patient. The SF-6D is a recent instrument that has not been used much yet, but it richer classification system could make it a more sensitive utility measure than the EuroQol measure.

3) Sciatica Frequency and Bothersome Index (SFBI). This is a scale from 0 to 6, which can assess the frequency (0 = not at all to 6 = always) and bothersomeness (0 = not bothersome to 6 = extreme bothersome) of back and leg symptoms. The sum of the results of four symptom questions yields both indexes, ranging from 0 to 24: leg pain; numbness and/or tingling in the leg; weakness in the leg or foot; pain in the back or leg while sitting. [17].

5) PROLO-scale. This scale measures the evaluation of the research nurse of the functional-economic status of the patients. This parameter has been used in studies on the difference in functional outcome between different techniques of lumbar spine fusion [40].

6) VAS pain in the back. This parameter measures the intensity of the pain in the back experienced during the week before visiting the research nurse. Assessment will be based on a horizontal 100 mm scale varying from 0 mm, 'no pain in the back', to 100 mm, 'the worst pain ever'. Patients do not see the results of earlier assessments and will score their pain during the visit. This parameter is included because a lot of patients with LSRS also have back pain in varying intensities, which can change after surgery or conservative treatment.

#### Other outcome measures

1) Costs. The societal costs during the first year will be estimated in accordance with the recent pharmacoeconomic guideline [47,48]. The costs of hospital admission and surgery will be based on an integral top-down cost analysis in three large regional participating hospitals (aggregated according to the total number of patients per department). From this institutional analysis, the constant costs per admission and the variable costs per admission day will be estimated. From these constant and variable costs, the individual costs of hospital admission and surgery for all patients can be estimated, using the duration of the hospitalization. In the study an MRI is performed in all cases. The costs of this MRI will only be calculated for patients undergoing surgery, because in the normal situation MRI would only be performed when a surgical indication exists.

Patients will register other health care needs in a diary (including physiotherapy, visits to GP's and specialists, nursing care and medication). Each diary covers a period of 3 months and will be discussed with the patient during the follow-up visits to the research nurse. The volume of health care will be assessed using standard prices [48].

In the diary the patient will also register direct non-medical costs (including time costs, travel expenses and domestic help). To estimate productivity costs the patients will also report absenteeism in the diary. At the follow-up visits, the research nurse will register the work situation, work efficiency and gross wages. Absenteeism will be valued according to the friction-cost method.

2) Incidence of (re-) surgery. One of the goals of the policy for group B is to avoid surgery while achieving at least the same effects. The surgical rate is therefore an indication of the success or failure of this policy. The incidence of re-operation at the same disc level in group A will be an indication of the failure rate for surgery.

3) Side-effects or complications that are ascribed to the treatment are recorded by the patients, their treating physicians and the research nurses.

4) MRI findings. The results of the differences between the baseline MRI and the MRI made 52 weeks after randomization are important secondary outcome measures. The difference in size of the disc herniation (in mm), nerve root compression, and amount of scar tissue will be registered. Failures of surgery can be recognized by inadequate disc removal or decompression of the nerve. The data will be gathered, using a standardized CRF, which will be filled out by the local radiologist, orthopaedic- or neurosurgeon and (neuro-) radiologist

### Sample size

The result of this study is based on the short-term success of surgical intervention and will be a trade-off between a quicker relief of leg pain versus an advantage in cost-effectiveness for conservatively managed patients. The sample size is calculated on the basis of the Roland Disability Questionnaire for Sciatica averaged during the 12 months follow-up period. The numbers used for this sample-size are drawn from the Maine Lumbar Spine Study 1 year and recently published 5-year results [19,55]. The difference in the Roland score between the surgical- and non-surgical group in this study did not change between 3 and 12 months follow-up as shown in their study [19] and can be averaged over the first year. The main aim of this study is to measure the short-term functional difference at 12 months follow-up. Surgical treatment is considered better when the post treatment change is at least 4 points more when compared to the conservative treatment arm [38] and constant over time. Considering this constant difference and a mean standard deviation =10 over the first year [55] 140 patients per treatment arm are needed to reach a power (1-β) of 0,90 with α = 0.05 (two-sided). To answer the main research question 280 patients are needed for analysis with at least 12 months follow-up. The aim is to enrol 300 (150 per arm) patients in the study, including 8 % loss to follow-up after 1 year. The total number of operated patients each year in all participating hospitals exceeds 1400. With this number of patients also a clinically important difference in median time to recovery of two months can be detected by survival analysis. Although the time to recovery is the main issue, the problem of recurrent complaints is still not solved in the different approaches of survival and proportional hazard analysis.

### Statistical and cost analysis

Baseline comparability will be investigated by descriptive statistics to examine if randomisation was successful. Differences in success rates between both groups are calculated, together with 95 per cent confidence intervals. In addition to an analysis of the difference in recovery between the two groups (as explained under the paragraph sample size) analyses of the difference in time to recovery will be carried out. Due to lack of data in the literature we could not base our sample size calculations on these differences. Survival-analysis is used to calculate differences in median time to recovery. Continuous outcomes are evaluated as change scores (differences between baseline measurement and each follow-up measurement). Multivariable analyses are performed to adjust for the eventual differences between the groups at baseline in prognostic indicators. All the analyses are performed according to the intent-to-treat principle. An additional per protocol analysis is performed comparing patients in the wait-and-see group who received surgery with patients in the same group who had not and with patients in the surgery group. To compare the actual treatment sec instead of strategies an explorative analysis will be performed in subgroups off all patients who actually received surgery and who did not receive surgery in both groups. All patients who withdraw from the study are included in the analysis until the time of withdrawal.

The result of this study will be a trade off between the disadvantages of surgery (hospitalisation, reduced quality of life and costs) versus the possible advantages (earlier relief of pain and return to work). For that reason recovery, measured as an 11 point difference in score when compared to baseline (Roland Disability Questionnaire for Sciatica), is the clinically most relevant patient outcome. Quality of Life (SF-36) and perceived recovery are important to compare the reduced quality of life from surgery to the possibly prolonged pain from conservative therapy and also to be able to compare cost-effectiveness with that of other spine interventions. The EuroQol is important to obtain cost-utility ratio's that can be compared with those of a wide range of other interventions. Utilities are obtained from the descriptive classification system of the EuroQol, using the model described by Dolan [43,53]. Conservative treatment may decrease costs compared to surgery but possibly at the expense of delayed effectiveness. In an incremental cost-effectiveness analysis, societal costs during the first year will be compared to the primary outcome measure (Roland Disability Questionnaire for Sciatica, averaged over the first year), Quality of Life (SF-36, during the first year) and perceived recovery (7-points Likert scale). Cost-effectiveness analyses with these effectiveness measures have been conducted before, allowing comparison with other spine interventions.

Finally, to answer the second research question explorative analyses are conducted to investigate whether the treatment effect after two, six and twelve months varies in specific subgroups of patients (Table 3).

**Table 3:** Selected prognostic variables for subgroup analysis

**Demographic Variables**
• Age < 39 years versus > 39 years,
• Intellectual versus physical demanding job,

**Anamnestic and Neurological Variables**
• Acute start LSRS versus slow start,
• History of backpain versus no history,
• Influence of coughing, sneezing on complaints versus no influence,
• Difficulty to put on shoes and/or socks versus no difficulty,
• Straight leg raising ≤ 30 degrees versus > 30 degrees,
• Positive crossed straight leg raising sign versus negative sign,
• VAS-pain > 70 versus < 69 mm,
• Tingling/numbness in pain area versus no tingling (9),
• Pain leg worse by sitting versus no worsening (9),
• McGill affective high score versus low score,

**Radiological Variables**
• MRI disc sequester versus contained disc herniation,
• MRI circumferential gadolinium enhancement versus no enhancement of disc herniation,
• Mediolateral versus median and lateral disc herniation,
• High versus low height of disc level (height 9 mm),

**Miscellaneous Variables**
• Preference for surgery versus no preference for surgery.
• Disc Herniation at L5S1 vs. L4L5

Using logistic regression for success rate and linear regression for severity of the disability, each prognostic indicator is checked for interaction with treatment. If the interaction term is significant, a stratified analysis will be performed.

## Discussion

In this article the rationale and design of a pragmatic RCT on the cost-effectiveness of timing of disc surgery for LSRS is described. The only randomized trial [7] so far on this subject only included patients where the caregiver was in doubt about the surgical indication. Patients with severe disabling pain were not randomized [8]. The Sciatica Trial is directed to those patients with a clear surgical indication according to current usual care. The study is pragmatic because it acknowledges that sometimes it may not be possible to postpone surgery for every conservative care patient until 6 months after allocation and that some patients will recover before surgery is performed in the surgical group. In these cases we consider it unethical to hold on to the randomized treatment. Because of the Intent-to-Treat analysis these cases will be analysed in their own allocated randomization arm and will not cause methodological problems because it is two healthcare strategies that are compared, as opposed to two treatments. The objective of this trial is to provide evidence on the preferred timing of disc surgery for sciatica. A prolonged conservative treatment strategy is compared to the international guideline advise of surgery after 6–8 weeks LSRS. The intended size of the study population is sufficiently large to detect short and long term differences between both strategies.

## Abbreviations

GP = General Practitioner

LSRS = Lumbosacral Radicular Syndrome

RCT = Randomized Controlled Trial

VAS = Visual Analogue Scale

## Competing Interests

The author(s) declare that they have no competing interests.

## Author's contributions

WP designed the study is responsible for the protocol. HH is responsible for the calculation of the sample size and contributed to the design of analysis. WH is responsible for the design of the cost-effectiveness analysis. RB has contributed in the case record forms and is responsible for the database ProMIse. JE contributed to the involvement of the GP's. JT structured the ideas about the diagnostics of LSRS and intake by neurologists. RT is the neurosurgical supervisor of WP. BK is the epidemiological supervisor of WP. All authors participated in the trial design and coordination. All authors read and approved the final manuscript.

## Pre-publication history

The pre-publication history for this paper can be accessed here:


